# Evaluating the Impact of rs4025935, rs71748309, rs699947, and rs4646994 Genetic Determinants on Polycystic Ovary Syndrome Predisposition—A Case-Control Study

**DOI:** 10.3390/life15040558

**Published:** 2025-03-29

**Authors:** Reema Almotairi, Rashid Mir, Kholoud S. Almasoudi, Eram Husain, Nabil Mtiraoui

**Affiliations:** 1 Department of Medical Laboratory Technology, Faculty of Applied Medical Sciences, Prince Fahad Bin Sultan Chair for Biomedical Research, University of Tabuk, Tabuk 71491, Saudi Arabia; kalmasoudi@ut.edu.sa (K.S.A.); e.husain@ut.edu.sa (E.H.); 2 Laboratory of Human Genome and Multifactorial Diseases, Faculty of Pharmacy, University of Monastir, Monastir 5000, Tunisia; mtiraouinabil@yahoo.fr

**Keywords:** polycystic ovary syndrome (PCOS), gene polymorphism, inflammatory markers, GSTM1 rs4025935 and GSTT1 rs71748309, ARMS-PCR-amplification-refractory mutation system PCR, IR-insulin resistance, Homeostatic Model Assessment for Insulin Resistance

## Abstract

**Background:** As a complicated endocrine condition, polycystic ovarian syndrome affects around 20% of women who are of reproductive age. It is linked to an increased risk of endometrial cancer, cardiovascular diseases, mental illnesses, non-alcoholic fatty liver disease, metabolic syndrome, and Type 2 diabetes. Despite numerous genetic studies identifying several susceptibility loci, these only account for approximately 10% of the hereditary factors contributing to PCOS, leaving its etiology largely unknown. While genome-wide association studies (GWAS) have been conducted on various populations to identify SNPs linked to PCOS risk, no such study has been reported in Tabuk. Thus, this study aims to investigate the association of a glutathione S-transferase M1 (GSTM1) deletion, VEGF gene (I/D) insertion/deletion, and VEGF-2578 gene polymorphism with polycystic ovarian syndrome. **Methodology:** In this research study (case-control), we utilized the ARMS-PCR to determine and analyze the polymorphic variants of VEGF-2578 C/A (rs699947). We employed multiplex PCR for the GSTM1 deletion and MS-PCR (mutation specific PCR) for the vascular endothelial growth factor gene insertion/deletion. **Results:** The findings indicated statistically significant differences in various biochemical and endocrine serum biomarkers, including lipid profiles (cholesterol, HDL, and LDL), Type 2 diabetes markers (HOMA-IR (Homeostatic Model Assessment for Insulin Resistance), free insulin fasting glucose), and hormone levels (testosterone, LH, progesterone and FSH) in PCOS patients. Specifically, regarding the GSTT1 genotype, individuals with the GSTT1-null genotype had an odds ratio (OR) of 4.16 and a relative risk (RR) of 2.14 compared to those with the GSTT1 genotype, with statistically significant differences (*p* = 0.0001). However, for the GSTM1 genotype, there was a statistically significant difference (*p* = 0.0002) in the OR and RR for the GSTM1-null genotype, which were 2.66 and 1.64, respectively. Protective effects were observed for individuals with either GSTT1 (+) GSTM1 (−) or GSTT1 (−) GSTM1 (+) genotypes, as well as for those with both null genotypes, yielding an OR of 0.41 and *p* < 0.003. The VEGF rs699947 C>A gene variation showed a statistically significant association between PCOS patients and controls (*p* < 0.020), with the A allele frequency higher among PCOS patients (0.42 vs. 0.30). Similarly, the VEGF rs4646994 I>D gene variation exhibited a statistically significant difference (*p* < 0.0034), with the D allele being more frequent in PCOS patients (0.52 vs. 0.35). The VEGF-A allele was strongly linked to PCOS susceptibility in the allelic model, exhibiting an OR of 1.62, RR of 1.27, and *p* < 0.007, while in the allelic comparison, the OR was 1.71, the RR was 1.32, and *p* < 0.004. **Conclusions:** This study concluded that null genotypes at rs4025935 and rs71748309, an insertion deletion at rs4646994, and the A allele of rs699947 were significantly associated with PCOS predisposition in our population and these could serve as potential loci for PCOS predisposition. To the best of our knowledge, it is the first study to highlight the association between these genetic variations and the predisposition of PCOS in our populations. Large-scale case-control studies in the future are required to confirm these results.

## 1. Introduction

The term PCOS was initially used in 1935 by Stein and Leventhal, who discovered a group of women with enlarged ovaries with many cysts, hirsutism, and amenorrhea [1].

PCOS is now understood to be a prevalent, diverse, and heritable endocrine condition that impacts women who are of reproductive age and contributes significantly to female infertility [2,3].

Current data indicate that about 20% of women aged 18 to 44 years are affected by this condition [4]. The clinical features associated with PCOS include acanthosis nigricans, irregular menstrual cycles, hyperandrogenism (such as hirsutism), and often subfertility [5]. The disorder has a multifactorial nature, characterized by a variety of clinical and biochemical phenotypes [6,7]. While the genetic causes of PCOS are not completely understood, numerous studies suggest a strong familial component and several genes that may contribute to susceptibility to the disease. For instance, genes involved in the biosynthesis of steroid hormones and the regulation of insulin action and secretion are implicated, especially since hyperinsulinemia is a prominent feature of PCOS [8]. Moreover, single-nucleotide polymorphisms (SNPs) are reported to play a crucial role in the etiology of PCOS [9,10]. Additionally, oxidative stress is another factor that can influence the pathophysiology of PCOS, serving as a biomarker for early diagnosis and screening in high-risk groups [11,12,13].

Glutathione S-Transferase Enzymes (GST) and PCOS. During oxidative stress, a significant amount of reactive oxygen species is generated and detoxified by a family of glutathione S-transferase enzymes (GST). Single-nucleotide polymorphisms in the GST genes may alter enzyme functions by decreasing their detoxification activities, ultimately increasing disease susceptibility [14]. Environmental factors, such as obesity, are also commonly observed in women with PCOS, with reports indicating that 40.5–80.5% of females are overweight or obese [15]. Other environmental modifiers, including infectious agents, diet, ethnicity, and toxins, may further contribute to the development and progression of PCOS [16,17]. PCOS early diagnosis is based on the Rotterdam Criteria, which requires the presence of two diagnostic indicators: anovulation/chronic oligo, hyperandrogenism, or polycystic ovaries identified through an ultrasonographic examination [18].

Vascular Endothelial Growth Factor Gene Insertion/Deletion (I/D) in PCOS. Follicle formation, ovulation, and the following expansion and regression of the corpus luteum (CL) all depend on ovarian angiogenesis [19]. Indeed, the function of the ovary relies heavily on the establishment and stabilization of blood vessels, which facilitate the delivery of nutrients, oxygen, and hormonal support to the follicles and corpus luteum (CL), mediating the release of steroids [20]. Vascular endothelial growth factor (VEGF) is a powerful cytokine with several functions, it has been extensively studied in the literature, and multiple lines of evidence show that VEGF is crucial for the physiological regulation of ovarian angiogenesis, remodeling of the vascular system, and the regression and function of the corpus luteum (CL) [17]. This implies that dysregulation of the VEGF gene increases the risk of PCOS development as it contributes to the oligoovulation or chronic anovulation, infertility, and OHSS (ovarian hyperstimulation syndrome). These are all common manifestations reported for PCOS [21,22,23]. In addition to VEGF, several other angiogenic factors, such as angiopoietins (e.g., Ang-1 and Ang-2), PDGF (platelet-derived growth factor) [24], TGF-β (transforming growth factor-β), and basic bFGF (fibroblast growth factor) are also dysregulated in PCOS [25].

VEGF-2578-rs699947C/A Polymorphism and PCOS.

The chromosomal region 6p21.3 contains the highly polymorphic VEGF gene. It has seven introns and eight exons, and alternative splicing produces a family of proteins [12]. The VEGFA gene’s promoter region at position -2549 has an 18-base pair insertion/deletion (I/D) polymorphism (rs35569394). Gene expression is impacted by this change, with allele D (deletion) having higher transcriptional activity than allele I (insertion) [26]. Numerous gynecological problems, such as recurrent spontaneous abortion [27], pre-eclampsia [28], uterine leiomyoma [29], and breast cancer susceptibility [30], have been linked to this genetic variation. Nevertheless, there is no published information on the rs35569394 polymorphism in PCOS according to current bibliographic reviews. Despite this, it is known that VEGF polymorphisms contribute to the etiology of PCOS, an endocrine metabolic disorder, and that individuals with PCOS have elevated VEGF protein expression [31]. The TGC haplotype may be linked to protective factors, while the VEGF gene rs1570360 polymorphism is linked to PCOS, according to recent research that our lab has performed. A meta-analysis of 29 case-control studies of 11 VEGF gene polymorphisms suggests that these genetic differences might become early predictors of PCOS [32,33].

## 2. Methodology

### 2.1. Study Participants and Criteria

Despite its complicated etiology, PCOS is typically diagnosed when two of the three symptoms are present: excess testosterone, ovulatory failure, and MOC-multiple ovarian cysts. Due to the disorder’s complexity, it is essential to evaluate clinical, endocrine, and ultrasound data simultaneously. Cases of PCOS were verified using the criteria established by the 2003 Rotterdam guidelines [18]. All cases were collected from the OPD (outpatient department) of the Obstetrics Unit at KSMH in Tabuk, Saudi Arabia. The study enrolled 125 clinically diagnosed polycystic ovary syndrome (PCOS) patients and 125 age- and gender-matched controls.

Inclusion and exclusion criteria of cases: This study included clinical cases of PCOS, and the population consisted of Saudi women only. The exclusion criteria included non-Saudis and expatriates.

Inclusion and exclusion criteria controls: The healthy group included gender- and age-matched healthy controls. The study excluded non-Saudis and expatriates. The biochemical serum profiles of the healthy controls included fasting glucose, HbA1c, insulin levels, serum lipids, and hormones. Fasting glucose levels were determined.

Blood specimen collection from PCOS/Controls:

Following an overnight fast, 3 mL of peripheral blood was extracted from each PCOS patient and gender-matched healthy controls. The specimen was placed into a red-top tube without the use of any anticoagulants. About 2 ml of the specimen was sent to the molecular biology lab, and the remaining was used for biochemical serum profiles. A single serum sample was promptly kept at −20 °C until the lipid profile was estimated. The second serum sample was sent out right away for biochemistry analysis.

### 2.2. Biochemical Serum Profile: For the First Phase, We Determined Biochemical Serum Profiles of the Patients, Such as HbA1c, Fasting Glucose, Insulin, Serum Lipids, and Hormones

Using a hexokinase kit (Cobas Integra 800; Roche, Munich, Germany), fasting glucose levels were measured. According to the vendor’s instructions, total insulin levels were tested using an ELISA kit (DRG-EIA). A HOMA calculator was used to determine the HOMA-IR index (https://www.dtu.ox.ac.uk/homacalculator/ accessed on 20 December 2024). Serum levels of many hormones, such as TSH, FSH, LH, estradiol, testosterone, and progesterone, were assessed using ELISA kits that were especially standardized for each assessment. Total cholesterol, triglycerides, LDL, and HDL levels in serum were determined through a colorimetric analysis (Integra 800; Roche). 

### 2.3. Extraction and Qualitative Assessment of Genomic DNA

Using a DNA extraction kit and the vendor’s instructions (Cat # 69506/Qiagen, Hilden, Germany), we isolated DNA from blood specimens of both PCOS patients and gender-matched healthy controls. The extracted DNA was kept for subsequent use at 4 °C after being dissolved in nuclease-free water. A NanoDrop spectrophotometer (Thermo Scientific, Waltham, MA, USA) was utilized to quantify the DNA, and an optical density ratio of A260 nm/A280 nm (1.65–1.97) was measured to qualitatively assess the extracted DNA.

### 2.4. Genotyping of VEGF rs4646994 I/D, VEGF rs699947 C/A, GSTM1 rs4025935, and GSTT1 rs71748309 Genes

Genotyping for the VEGF rs699947 C/A mutation was determined by using ARMS-PCR, while the VEGF rs4646994 D>I mutation was determined by MS-PCR. Using multiplex PCR, genotyping for GSTT1 and GSTM1 (rs4025935 and rs71748309) was performed. The primers for the ARMS, MS-PCR, and multiplex PCR methods are shown in Table 1. The primers for rs699947 were designed using Primer 3.0 version, as detailed in Table 1.

### 2.5. Preparation of PCR Cocktail

For identification of the VEGF-rs699947 (-2578) C/A genotypes in samples from individuals with PCOS and control subjects, ARMS-PCR was employed. Primer 3.0 v software was utilized to design the rs699947 oligonucleotides, as depicted in Table 1. The ARMS-PCR reaction was conducted in a total volume of 12 ul, consisting of 55 ng of template DNA, 0.10 μL of forward primer (Fo), 0.12 μL of reverse primer (Ro), 0.12 μL of fluorescent probe (FI), 0.12 μL of reverse probe (RI) (25 pmol of each primer), and 6 μL of green master Mix Cat No M712C (Promega, Madison, WI, USA). The final volume was adjusted to 23 μL by adding nuclease-free water and 2 μL of DNA from each patient.

### 2.6. PCR Program

The PCR thermocycling conditions were as follows: 95 °C for 8 min, followed by 30 cycles of denaturation at 94 °C for 30 sec; annealing for 30 s at 58 °C for VEGF-rs699947, (62 °C), VEGF rs4646994 I/D (58.8 °C), for GSTT1 and GSTM1 (rs4025935 and rs71748309) (60 °C); initial extension at 72 °C for 45 sec, then extension (final) at 72 °C for 09 min and then stored at 4 °C.

### 2.7. PCR Product Visualization in Gels

Electrophoresis on a 2% agarose gel was used to separate the PCR products, which were then stained with SYBR Safe dye and observed with a UV transilluminator manufactured by Bio-Rad in Hercules, CA, USA.

### 2.8. VEGF-rs699947 (-2578) C>A Genotyping

**Figure 1:**
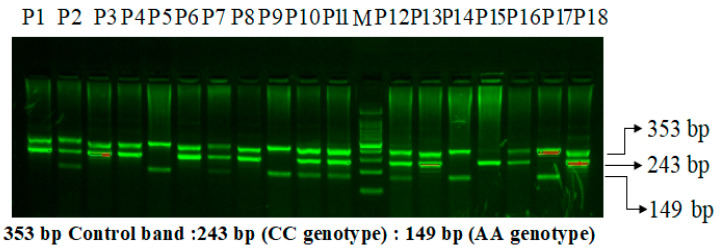
Primer optimization for the VEGF-2578 C>A genotyping in PCOS patients using the amplification-refractory mutation system (ARMS). M: 100 bp DNA ladder. Heterozygous-(AC): P2, P7, P10, P11, P12, P16. Homozygous-(AA): P5,P9,P14,P17. Homozygous-(CC): P1, P3, P4, P6, P8, P13, P15, P18.

On a 2% agarose gel, the VEGF-2578 C>A gene amplification products were separated via electrophoresis, stained with 0.5 μg/mL ethidium bromide, and observed using a UV transilluminator. The VEGF-2578 C>A gene’s exon is flanked by the primers FO and RO, which provide a 353 bp control band to measure the amount and quality of DNA. The primers Fwt and RO amplify the wild-type allele (C genotype) in Figure 1, resulting in a 243 bp band, while the primers FO and Rmt amplify the mutant allele (A genotype), resulting in a 149 bp band.

**Figure 2:**
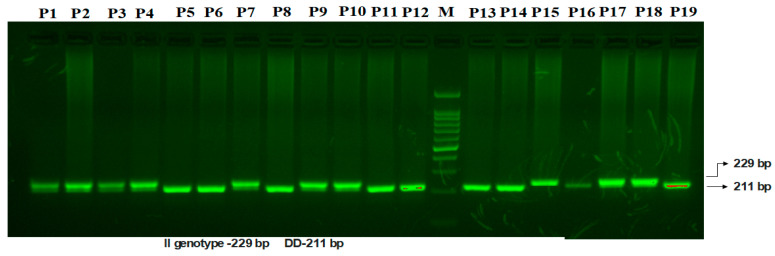
Amplification of mutation-specific PCR for (I/D) VEGF promoter region 18-bp polymorphism determination in PCOS patients. M: 100 bp DNA ladder. Heterozygous-(I/D): P1, P2, P3, P4, P7, P9, P10. Homozygous-(DD): P5, P6, P8, P11, P12, P13, P14, P16, P19. Homozygous-(II): P15, P17, P18.

**VEGF I/D Polymorphism.** The 18 kb fragment involved in the insertion/deletion (I/D) polymorphism is situated at the −2549 location of the VEGF gene promoter region. Figure 2 illustrates the two bands found for the VEGF I/D polymorphism: a 211 bp band for the D allele and a 229 bp band for the I allele.

#### Multiplex PCR for GSTT1 and GSTM1 (rs4025935 and rs71748309) Genotyping

**Figure 3:**
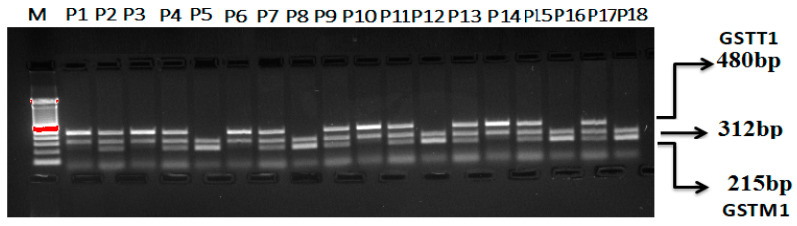
Optimization of multiplex PCR for GSTT1 and GSTM1 (rs4025935 and rs71748309) genotyping. M: 100 bp DNA ladder. GSTT1(+): P1, P3, P6, P10, P14. GSTM1(+): P5, P8, P12, P16, P18. GSTM1+GSTT1: P2, P4, P7, P9, P11, P13, P15, P17. GSTM1 (deletion): P1, P3, P6, P10, P14. GSTT1 (deletion) (+): P5, P8, P12, P16, P18.

To ascertain both GSTM1’s and GSTT1′s allelic status (rs4025935 and rs71748309) concurrently, a multiplex PCR assay was optimized. This assay amplified gene segments for GSTM1 and GSTT1, along with fragments specific to the deletions in these genes, using four primers. The deletions in GSTM1 and GSTT1 are believed to result from unequal crossing over between two highly homologous regions flanking these genes. To enhance the robustness of the multiplex PCR, the amplification lengths were kept as short as possible. The optimized multiplex PCR achieved balanced reactions that included all possible combinations of the amplicons. Both positive genotypes for GSTM1 and GSTT1 are indicated by the presence of two bands: 215 bp and 480 bp. Figure 3 demonstrates that no bands were observed for genotypes lacking both GSTM1 and GSTT1. Bands of 215 bp suggest genotypes that lack GSTM1, while bands of 480 bp indicate genotypes that lack GSTT1.

**Statistical Analysis.** For continuous variables, group differences were evaluated using the student’s two-sample *t*-test or one-way analysis of variance (ANOVA); for categorical variables, including deviations from the Hardy–Weinberg equilibrium (HWE), the Chi-squared (χ^2^) test was used. Allelic and genotypic frequencies for VEGF rs4646994 I/D, VEGF rs699947 C/A, GSTM1 rs4025935, and GSTT1 rs71748309 were evaluated using the Chi-square test. The associations between VEGF C/A, VEGF D/I, GSTM1 rs4025935, and GSTT1 rs71748309 genotypes and cases of PCOS were assessed through estimations of odds ratios (ORs), risk ratios (RRs), and risk differences (RDs) with 95% confidence intervals (CIs). A *p*-value of <0.05 was considered significant. GraphPad Prism 8.4 and SPSS 16 were used to conduct all statistical analyses.

## 3. Results

***Hardy–Weinberg Equilibrium (HWE).*** This research investigation has demonstrated that the allele frequencies for VEGF-2578 C/A (rs699947), VEGF I/D (rs4646994), GSTM1 (rs4025935), and GSTT1 (rs71748309) among the control subjects comply with the Hardy–Weinberg equilibrium (HWE). The genotype distributions and allele frequencies of the SNPs situated in VEGF-2578 C/A (rs699947) indicated no deviation from the HWE in the matched healthy control group (*p* > 0.05) with an χ^2^ value of 0.55 (*p* ≤ 0.45). Similarly, the genotype distributions and allele frequencies for VEGF I/D (rs4646994) also showed no deviation from the HWE (*p* > 0.05) with an χ^2^ value of 0.121 (*p* ≤ 0.289). To validate the results of genotyping, it was shown that 10% of samples from the normal control group were randomly selected, revealing an accuracy rate of over 99%. Similar findings were observed for GSTM1 (rs4025935) and GSTT1 (rs71748309).

***Demographic Characteristics of the Study Population.*** The study included 250 participants, with 125 diagnosed with polycystic ovary syndrome (PCOS) and 125 matched healthy controls. Due to the complexity of PCOS, its effects are reflected by various clinical biomarkers that are significantly altered in patients. Table 2 presents the demographic characteristics of both the PCOS patients and the controls. Compared to healthy controls, most of the tested biomarkers in PCOS patients showed significant differences, as indicated in Table 2. With the progression of the disease, patients exhibited increased fasting glucose and insulin levels, suggesting the development of Type 2 diabetes mellitus (T2DM) and insulin resistance.

At the time of study inclusion, the mean age of the PCOS patient group was approximately 28.55 years, while the control group had a mean age of 29.20 years. Most biochemical markers examined in individuals with PCOS revealed significant variations. The lipid profile indicated higher levels of serum cholesterol, triglycerides (TAGs), low-density lipoprotein (LDL), and high-density lipoprotein (HDL) in PCOS patients compared to the control group. Additionally, levels of progesterone, luteinizing hormone, and follicle-stimulating hormone showed significant differences in the patient group. Higher testosterone levels were observed in PCOS patients exhibiting hyperandrogenism, which is a defining characteristic of this endocrine and metabolic disorder. The altered lipid profiles in patients were associated with significant differences in their mean body mass index (BMI).

The potential association of GSTM1-rs4025935 and GSTT1-rs71748309 genotypes with susceptibility to PCOS. The findings indicated that 30 (24%) of the control group and 71 (56.8%) of the case group had the GSTT1 (−) genotype.

For GSTM1(−), the frequencies were 75 (60%) in the PCOS case group and 45 (36%) in the control group. Both GSTT1- and GSTM1-null genotypes were somewhat higher in the case group than in the control group, with 42 (33.6%) and 25 (20%), respectively (see Table 3). A multivariate analysis using logistic regression was conducted to determine the correlation between GSTT1 (+) and GSTT1 (−) genotypes and susceptibility to PCOS in the Tabuk population. Regarding the GSTT1 genotype, it was reported that the odds ratio (OR) and relative risk (RR) for the GSTT1-null genotype, compared to the presence of the GSTT1 genotype, were estimated at 4.16 (2.42 to 7.16) and 2.14 (1.55 to 2.97), respectively, with statistically significant differences (*p* = 0.0001; see Table 3). There was a statistically significant difference (*p* = 0.0002) in the OR and RR for the GSTM1 genotype and the GSTM1-null genotype, which were calculated to be 2.66 (1.60 to 4.45) and 1.64 (1.26 to 2.15), respectively. Furthermore, there was a protective effect observed with the combinations of GSTT1 (+) GSTM1 (−) and GSTT1 (−) GSTM1 (+), as well as both null genotypes in relation to PCOS patients, with an OR of 0.41 (0.19 to 0.93) and RR of 0.62 (0.42 to 0.94), yielding a significance level of *p* < 0.003.

The allele and genotype frequencies of the VEGF rs4646994 I/D and VEGF rs699947 C/A gene polymorphisms were analyzed in both PCOS cases and control groups. For the VEGF rs4646994 I/D genotypes, the frequencies were as follows: in the PCOS cases, II was 25.5%, ID was 40%, and DD was 32%. In the control group, the frequencies were II at 30.4%, ID at 53.6%, and DD at 16% (see Table 4). The variation in the VEGF rs4646994 I/D gene between PCOS patients and controls was statistically significant (*p* < 0.0034). Additionally, the frequency of the D allele was higher among PCOS patients than among controls (0.52 vs. 0.35). Regarding the VEGF rs699947 C/A gene polymorphism, the frequencies were as follows: in PCOS cases, CC was 36%, CA was 43.81%, and AA was 20.8%. In controls, the frequencies were CC at 50.4%, CA at 39.2%, and AA at 10.4% (see Table 4). The variation in the VEGF rs699947 C/A gene between PCOS patients and controls was also statistically significant (*p* < 0.020). Moreover, the frequency of the A allele was higher among PCOS patients than among controls (0.42 vs. 0.30) (see Table 4).

Logistic Regression Analysis of VEGF rs4646994 I/D Genotypes to Predict the Risk of PCOS Susceptibility. The VEGF-ID genotype or heterozygosity was not associated with susceptibility to PCOS, as indicated by an odds ratio (OR) of 0.88 (95% CI: 0.4883 to 1.6084), a relative risk (RR) of 0.94 (95% CI: 0.7266 to 1.2368), and a *p*-value of 0.69. Conversely, the DD genotype showed a strong association with PCOS susceptibility, with an OR of 2.55 (95% CI: 1.2566 to 5.1874), an RR of 1.71 (95% CI: 1.1223 to 2.6054), and a *p*-value of 0.009 (see Table 5). In the dominant inheritance model, there was no association between the VEGF-(ID+DD) genotypes and the VEGF (II) genotype, evidenced by an odds difference (OD) of 1.26 (95% CI: 0.7297 to 2.2084), an RR of 1.12 (95% CI: 0.8636 to 1.4606), and a *p*-value of 0.390. However, a significant association was found between the VEGF-(II+ID) genotypes and the VEGF-(D) genotype regarding PCOS susceptibility. This was supported by an OR of 2.75 (95% CI: 1.5048 to 5.0367), an RR of 1.76 (95% CI: 1.2052 to 2.5957), and a *p*-value of less than 0.001 in the recessive model. Additionally, in the allelic comparison, the VEGF-A allele was strongly associated with PCOS susceptibility, with an OR of 1.62 (95% CI: 1.1390 to 2.3121), an RR of 1.27, a CI of 1.0653 to 1.5226, and a *p*-value of less than 0.007 (Table 5).

Logistic Regression Analysis of VEGF rs699947 C>A variants to estimate the PCOS Susceptibility. Our results indicated that in the codominant model, the heterozygous VEGF CA genotype was not significantly correlated with the risk of PCOS, showing an odds ratio (OR) of 1.54 (95% CI = 0.8955 to 2.6580), relative risk (RR) = 1.22 (95% CI = 0.9474 to 1.5869), and *p* < 0.11. Conversely, the VEGF -AA genotype exhibited a strong association with PCOS susceptibility, presenting an OR of 2.80, RR of 1.75, and *p* < 0.008 in the codominant inheritance model (Table 6). In the dominant model, the comparison of VEGF (CA+AA) versus the CC genotype revealed an increased susceptibility to PCOS, with an odds ratio of 1.80 (95% CI = 1.0888 to 2.9971), RR = 1.33 (95% CI = 1.0451 to 1.7079), and *p* < 0.022. Additionally, a significant association was found between the VEGF (CA+CC) versus VEGF AA genotypes and PCOS susceptibility, indicated by an OR of 2.26 (95% CI = 1.1029 to 4.6418), RR = 1.59 (95% CI = 1.0036 to 2.5266), and *p* < 0.025 in the recessive model. Furthermore, the VEGF-A allele was associated with PCOS susceptibility in the allelic comparison, showing an odds ratio of 1.71 (95% CI = 1.1877 to 2.483), RR = 1.32, and *p* < 0.004 (Table 6).

## 4. Discussion

Cardiometabolic conditions, including obesity, insulin resistance, and Type 2 diabetes mellitus, are more common in women with PCOS, as are infertility and pregnancy difficulties. Furthermore, the quality of life in this population is greatly impacted by mental comorbidities, such as anxiety and depression. Despite the fact that obesity increases these health risks, the precise etiology and pathophysiology of PCOS are still unclear and complicated. Genome-wide association studies have identified genetic variations linked to numerous disorders, including PCOS [34,35].

**Figure 4:**
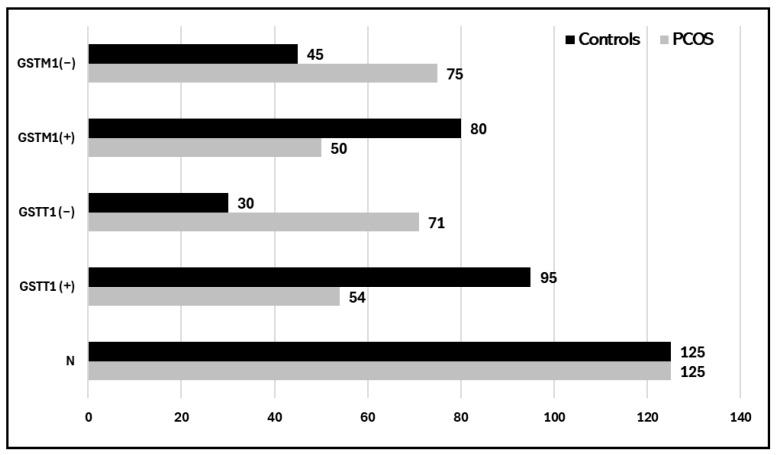
Histogram showing the distribution of the GSTM1-rs4025935 and GSTT1-rs71748309 null genotypes among PCOS cases and controls.

Fort the comparative association of GSTM1-rs4025935 and GSTT1-rs71748309 null genotypes in PCOS patients, environmental and occupational factors, such as a sedentary lifestyle and poor dietary habits, play a crucial role in exacerbating PCOS. These factors can lead to irregular menstruation, weight gain, reduced physical activity, and disruptions of the menstrual cycle, all of which contribute to the prevalence and progression of PCOS [36]. Over time, manifestations of PCOS have been noted, including a 2–3 times increase in Anti-Mullerian hormone (AMH) levels in affected women. Levels of antimüllerian hormone (AMH) have been suggested as a substitute marker for PCOS and PCOM [37,38,39]. This study analyzes the pathophysiology of PCOS in the context of various genes involved in disrupted biochemical pathways. Glutathione S-transferase system 1 (GSTM1) and GSTT1, encoded by the mu (μ) and theta (θ) genes, are phase II multifunctional enzymes crucial for bioactivation and cellular detoxification processes. Epidemiological studies indicate that deletion polymorphisms of the GSTM1 and GSTT1 genes are prevalent in human populations and have been thoroughly investigated. These genes are crucial antioxidant enzymes implicated in steroidogenesis [40]. The frequency and distribution of the GSTM1-rs4025935 and GSTT1-rs71748309 null genotypes among PCOS cases and controls is depicted in Figure 4. The frequency of GSTM1 and GSTT1 polymorphisms varies considerably among different ethnic groups and even within the same ethnic population across different countries [41]. Thus, GST genes play a vital role in female reproduction, as they are found in high concentrations in the placenta and ovarian follicles. Research suggests that the null genotypes of GSTM1 and GSTT1 may elevate the risk of cervical cancer (CC) and ovarian cancer (OC) and are linked to factors associated with polycystic ovary syndrome (PCOS). Nonetheless, the outcomes of original investigations and meta-analyses have produced incongruous results. The presence of GSTT1 and GSTM1 null genotypes is associated with an increased vulnerability to infertility. Individuals possessing the GSTT1-null genotype seem to have a heightened susceptibility to developing polycystic ovary syndrome (PCOS) and infertility due to diverse etiologies moreover, the GSTT1 null genotype, whether alone or in combination with other factors, is associated with an increased risk of infertility development, independent of its underlying cause.

The GSTM1-null genotype is only associated with all causes of infertility when the GSTT1 is null [42]. A similar study conducted on 180 PCOS-afflicted women from south India found that the presence of a hyper-inducible CYP1A1 (T6235C) mutant genotype and its mutants in combination with GSTM1- and GSTT1-null genotypes may result in an imbalance between phase I and phase II enzymes and thus may be a risk factor for PCOS [43]. A combination AhRR (aryl hydrocarbon receptor repressor) CG or GG and the GSTT1-null genotype or a combined GSTT1/GSTM1-null genotype may also be linked to an elevated risk of PCOS, according to studies conducted on 478 Korean women [44]. Numerous studies have demonstrated a link between GSTM1 polymorphisms and disorders in both genders. Li et al. conducted a meta-analysis revealing that males with dual null genotypes of GSTM1/GSTT1 exhibit heightened susceptibility to idiopathic infertility among Caucasians, with the GSTM1-null genotype significantly increasing the risk of male idiopathic infertility [45]. These findings align with a meta-analysis conducted by Wu et al., which demonstrates the association between a GSTM1 polymorphism and male infertility in both Asian and Caucasian populations [46]. Nair et al. [47] reported, in their study and meta-analysis, a significantly elevated risk of recurrent pregnancy loss (RPL) linked to GSTT1 and GSTM1 polymorphisms; however, these findings cannot be generalized across diverse populations, as this association appears to be ethnicity-dependent. Conflicting investigations have suggested that the single deletion polymorphisms of the GSTT1 and GSTM1 genes are not linked to PCOS; nevertheless, their combination may be involved in the condition’s genesis [10]. A meta-analysis conducted by Makoui et al. demonstrated no correlation between GSTM1 and GSTT1 polymorphisms and an elevated risk of PCOS [48]. The authors unequivocally recommend additional evidence to substantiate the conclusions. This evidence suggests many factors affecting the disease’s etiology globally, necessitating a case-by-case investigation. Despite the significance of GSTM1 and GSTT1 gene variations and certain inconsistencies identified in prior research, there is currently a paucity of studies examining the relationship between the genetic polymorphisms of GSTT1 and GSTM1 and PCOS. This study aims to evaluate the interplay between genes and the environment in the course of PCOS within this particular demographic group.

### Comparative Association of VEGF rs699947 C>A Genotypes in PCOS Patients

A connection exists between angiogenesis and polycystic ovary syndrome (PCOS). Angiogenesis, the formation of new blood vessels, is essential for ovarian function and fertility. In PCOS, altered angiogenic activity can contribute to the development of ovarian cysts, disrupt normal follicle development, and affect hormone production. Studies have shown that women with PCOS often exhibit abnormal levels of angiogenic factors, such as vascular endothelial growth factor (VEGF). These imbalances can lead to increased ovarian hyperandrogenism and insulin resistance, both of which are key features of PCOS. Additionally, impaired angiogenesis may influence the overall metabolic health of individuals with PCOS, linking it to related conditions, like obesity and diabetes. Understanding the role of angiogenesis in PCOS could lead to new therapeutic strategies aimed at managing the symptoms and long-term health risks associated with the condition. VEGF single nucleotide polymorphisms (SNPs) are increasingly recognized for their potential role in polycystic ovary syndrome (PCOS), a complex endocrine disorder. These genetic variations may influence VEGF levels and activity, which are critical for ovarian function and the regulation of angiogenesis in ovarian tissues. Understanding the association between VEGF SNPs and PCOS can provide insights into the pathophysiology of the syndrome, particularly regarding ovarian follicle development and insulin resistance. Identifying specific VEGF SNPs linked to PCOS risks may also pave the way for personalized treatment strategies, enhancing management approaches for those affected by this condition. Several single nucleotide polymorphisms (SNPs) of the VEGF gene are associated with abnormal VEGF protein secretion.

**Figure 5:**
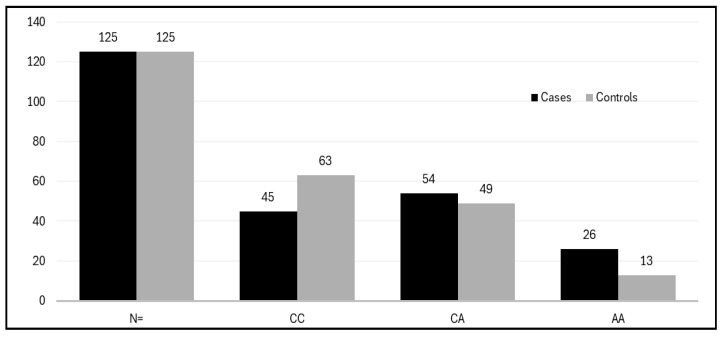
Histogram showing the distribution of the genotypes of the VEGF rs699947 A>C gene among cases and controls.

In our study, we investigated the association of the VEGF rs699947 A>C genotype with PCOS susceptibility. VEGF rs699947 A>C genotype frequencies in our PCOS cases and controls is depicted in Figure 5. Our results show a statistically significant association with PCOS patients, with a *p*-value of 0.02. It was revealed that PCOS patients had a greater frequency of the A allele (0.42 vs. 0.30) than did healthy control individuals, with *p* = 0.0041 (Table 4). In the codominant inheritance model, the VEGF -AA genotype was highly correlated with the likelihood of developing PCOS with an OR of 2.80, RR 1.75, and *p* < 0.008. This result aligns with the study by Huang and Wang (2020), who conducted a meta-analysis identifying 9 studies, including 29 case-control studies and 11 polymorphisms in VEGF, and found a significant increase in this particular genotype among PCOS patients [49]. On another hand, our results contradict a meta-analysis conducted by Li et al. (2020), who analyzed seven eligible studies involving 1100 PCOS patients and 1141 control individuals. Among other findings, they showed no significant association of rs699947 with PCOS for all women, regardless of ethnicity [50]. PCOS is closely linked to an increased risk of developing Type 2 diabetes mellitus, primarily due to insulin resistance, which is a common feature of PCOS. Women with PCOS often have elevated insulin levels, which can lead to higher blood sugar levels and an increased likelihood of developing diabetes over time. This connection is exacerbated by associated factors, such as obesity and metabolic syndrome, which are prevalent in many individuals with PCOS. Understanding this relationship is crucial for early intervention and management, as addressing insulin resistance through lifestyle changes or medication can help mitigate the risk of T2DM in affected women. VEGF SNPs have been extensively studied with T2DM to find any association with susceptibility to the disease. A study by Sing et al. (2022) aimed to investigate the association of the VEGF gene polymorphism rs699947 with diabetic retinopathy (DR) and showed that the C-allele (rs699947) was 1.66 times more prevalent in subjects with severe DR compared to diabetic controls with no retinopathy.

**Figure 6:**
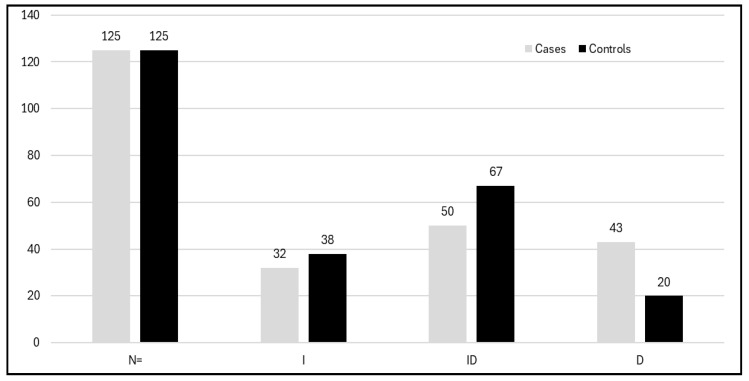
Histogram showing distribution of the genotypes of VEGF I/D polymorphism among cases and controls.

VEGF rs4646994 I/D: When linked with deletions/additions in VEGF, the DD-genotype was 2.73 times overexpressed in the severe DR category compared to subjects with no DR in the male subgroup. This is due to high linkage disequilibrium between the two polymorphisms [51]. The frequency of the VEGF rs4646994 I/D genotypes among PCOS cases and controls is depicted in Figure 6.

Another study was performed by Hu et al. (2021) to investigate the link between rs699947 and diabetic retinopathy. Analyses conducted by subgrouping according to ethnicity found that rs699947 is associated with non-proliferative diabetic retinopathy (NPDR) (dominant model: OR = 2.04, 95%CI = 1.30–3.21) in the total population and with PDR (dominant model: OR = 1.72, 95%CI = 1.05–2.84) [52] in Asians. The VEGF gene exhibits significant variability, with functional variations influencing the production of the VEGF protein. There is a functional 18 bp insertion/deletion (ins/del) polymorphism affecting gene expression at position −2549 in the VEGF promoter region. The del allele increases transcriptional activity 1.95 times more than the ins allele. A study by Elfaki et al. (2021) found that the VEGF rs699947 CA genotype and D allele of VEGF I/D polymorphisms were associated with T2DM, which suggests that they are potential risk loci for the development of T2DM [31]. A case-control model comparing 33 DR patients and 35 non-DR controls found significant genetic variation in the VEGF rs699947 C/A polymorphism, with the C allele being higher in the DR group. In addition, the C allele was found to be a risk factor for DR in T2DM patients in Bali, Indonesia. The study could help to manage DR patients earlier to avoid further complications [53].

Our study suggests that the VEGF gene polymorphism rs699947 A>C may be associated with an increased risk of PCOS, indicating that this biomarker could serve as an early detection tool. However, further investigation is necessary to establish rs699947 as a definitive marker for identifying individuals at a higher susceptibility to developing PCOS. In our study, all PCOS patients examined also had Type 2 diabetes mellitus (T2DM), making it challenging to determine whether the significant association observed is related specifically to the PCOS condition or to T2DM. Participants recruited for such research need to undergo thorough and detailed assessments due to the complexities of the disease and its relationship with various symptoms and health conditions. Additionally, different methodologies are required to provide supportive evidence of the association between VEGF SNPs and PCOS. New approaches, such as NGS analysis, may aid in uncovering stronger evidence. Future investigations into the relationship between VEGF SNPs and PCOS are expected to enhance our understanding of the genetic foundations of this complex condition. Researchers may prioritize large-scale genomic studies to identify additional SNPs linked to PCOS, which would improve our understanding of genetic risk factors. Including diverse populations in these studies will enhance the generalizability of the findings and help clarify population-specific variations. Furthermore, integrating transcriptomic and proteomic analyses can shed light on how VEGF SNPs influence ovarian functions and angiogenesis in the context of PCOS. Finally, exploring potential therapeutic interventions that target VEGF pathways could lead to novel treatments aimed at alleviating the reproductive and metabolic complications associated with PCOS.

## 5. Conclusions

This study concluded that null genotypes at rs4025935 and rs71748309, an insertion deletion at rs4646994 and the A allele of rs699947 were significantly associated with PCOS predisposition in our population and these could serve as potential loci for PCOS predisposition. To the best of our knowledge, it is the first study to highlight the association between these genetic variations and the risk of PCOS in our populations. Future extensive case-control studies are necessary to validate these findings.

## Figures and Tables

**Table 1:** ARMS-primers for VEGF-rs699947 C>A, multiplex PCR primers for GSTT1 and GSTM1, and mutation-specific PCR primers for the VEGFrs4646994 D>I gene variation.

ARMS-Primers for VEGF-rs699947 C>A					
Gene	Allele	Sequence	PCR Product	Annealing Temperature	References
VEGF Fo		5-CCTTTTCCTCATAAGGGCCTTAG-3	353 bp	58.1 °C	[20,30]
VEGF Ro		5-AGGA AGC AGCT TGGAA AAA TTC-3			
VEGF FI	A allele	5-TAGG CCA GAC CCTG GCAA-3	149bp		
VEGF RI	C allele	5-GTC TGA TTA TCCA CCC AGAT CG-3	243bp		
MS-Primers for VEGF rs4646994 I/D					
VEGF F	D-allele	5′-GCTGAGAGTGGGGCTGACTAGGTA-3′	211 bp	58.8 °C	[31]
VEGF R	I-allele	5′-GTTTCTGACCTGGCTATTTCCAGG-3′	229 bp		
GSTT1 and GSTM1 (rs4025935 and rs71748309) Multiplex PCR Primers for Genotyping					
GSTT1 F	GSTT1	5′-TTCCTTACTGGTCCTCACATCTC-3′	480 bp	60 °C	[14]
GSTT1 R		5′-TCA CGG GAT CAT GGC CAG CA-3′			
GSTM1 F	GSTM1	5′-GAA CTC CCT GAA AAG CTA AAG C-3′	215 bp		
GSTM1 R		5′-GTT GGG CTC AAA TAT ACG GTG G-3′			
CYP1A1F	CYP1A1	5′-GAA CTG CCA CTT CAG CTG TCT-3′	312 bp		
CYP1A1R		5′-CA G CTG C AT T T GG AA G T G C TC-3′			

**Table 2:** Laboratory investigation of lipid profiles and endocrine indicators between PCOS and control, as well as demographic data.

Characteristic	Controls ^J^	Cases ^J^	*p* ^K^
FBG (mmol/L) ^G^	4.60 ± 0.66	7.60 ± 5.16	<0.0020
Age ^Y^	28.55 ± 4.99	29.20 ± 6.10	0.420
BMI (kg/m2) ^G^	29.10 ± 4.05	27.11 ± 5.90	<0.004
HDL (mmol/L) ^G^	1.73 ± 0.89	1.78 ± 0.91	<0.0032
LDL (mmol/L) ^G^	2.98 ± 0.70	4.98 ± 1.90	<0.0024
Cholesterol (mmol/L) ^G^	3.80 ± 0.60	5.23 ± 0.81	<0.0023
Triglycerides (mmol/L) ^G^	1.78 ± 0.65	3.58 ± 1.41	<0.0471
Luteinizing hormone (mIU/mL) ^M^	0.09 (0.09–1.90)	3.65(0.75–9.10)	<0.0040
Testosterone (ng/dL) ^M^	14.90 (6.80–13.55)	60.15 (46.80–70.30)	<0.0040
Estradiol (pmol/L) ^M^	334.70 ± 249.49	386.66 ± 336.79	0.169
Progesterone (ng/mL) ^M^	15.55 (3.10–18.10)	18.30 (2.40–33.10)	<0.0040
HOMA-IR ^G^	4.00 ± 0.82	6.00 ± 5.88	<0.0030
Free Insulin (mU/mL) ^G^	7.90 ± 2.99	14.39 ± 5.97	<0.0021
FSH (mIU/mL) ^M^	0.89 (0.11–5.21)	4.10 (3.15–6.11)	<0.0040

Abbreviations: ^J^ 125 PCOS patients and 125 gender-matched healthy controls; ^K^ Student’s *t*-test for continuous variables, *p* value; ^G^ values as mean ± SD; ^M^ values presented as median (interquartile range); Mann–Whitney U-test (normally distributed variables); ^Y^ value as a mean.

**Table 3:** Frequencies [*n* (%)] of null genotypes of GSTT1 and GSTM1 in PCOS and control groups.

Variables				Controls (125)		PCOS (125)	
GSTT1 genotype frequency in case and control groups							
GSTT1 (+)				95 (76%)		54 (43.2%)	
GSTT1 (−)				30 (24%)		71 (56.8%)	
Genotype frequencies of GSTM1 in case and control groups							
GSTM1(+)				80 (64%)		50 (40%)	
GSTM1(−)				45 (36%)		75 (60%)	
Genotype frequencies of GSTT1/GSTM1 in case and control groups							
GSTT1 (−) GSTM1 (−)				42 (33.6%)		25 (20%)	
GSTT1 (+) GSTM1 (+)				33 (26.4%)		31 (24.8%)	
GSTT1 (+) GSTM1 (−)				30 (24%)		54 (43.2%)	
GSTT1 (−) GSTM1 (+)				20 (16%)		15 (12%)	
Association of GSTM1/GSTT1 null genotypes with PCOS risk							
Association of GSTT1 (+) and GSTT1 (−) genotypes with PCOS risk							
Variables	N = 125	N = 125	OR (95% CI)		RR (95% CI)		*p* value
GSTT1 (+)	95(76%)	54 (43.2%)	Ref. 1.00		Ref. 1.00		
GSTT1 (−)	30 (24%)	71 (56.8%)	4.16 (2.4215 to 7.1589)		2.14 (1.5532 to 2.9665)		0.0001
Association of GSTM1 (+) and GSTM1 (−) genotypes with PCOS risk							
Variables	N = 125	N = 125	OR (95% CI)		RR (95% CI)		
GSTM1 (+)	80 (64%)	50 (40%)	Ref. 1.00		Ref. 1.00		
GSTM1 (−)	45 (36%)	75 (60%)	2.66 (1.5992 to 4.4466)		1.64 (1.2552 to 2.1454)		0.0002
Association of GSTT1 (+) GSTM1 (+) and GSTT1 (−) GSTM1 (−) genotypes with PCOS risk							
Variables	N = 125	N = 125	OR (95% CI)		RR (95% CI)		*p* value
GSTT1 (−) GSTM1 (−)	42 (33.6%)	25 (20%)	Ref. 1.00		Ref. 1.00		
GSTT1 (+) GSTM1 (+)	33 (26.4%)	31 (24.8%)	1.57 (0.7863 to 3.1678)		1.08 (0.7959 to 1.4820)		0.19
Association of GSTT1 (+) GSTM1 (+) and GSTT1 (−) GSTM1 (−) genotypes with PCOS risk							
Variables	N = 125	N = 125	OR (95% CI)		RR (95% CI)		*p* value
GSTT1 (+) GSTM1 (+)	33 (21.81%)	31 (38.18%)	Ref. 1.00		Ref. 1.00		
GSTT1 (−) GSTM1 (−)	42 (29.09%)	25(30%)	0.63 (0.3157 to 1.2719)		0.82 (0.6088 to 1.1113)		0.199
Association of GSTT1 (+) GSTM1 (−) and GSTT1 (−) GSTM1 (−) genotypes with PCOS risk							
Variables	N = 125	N = 125	OR (95% CI)		RR (95% CI)		*p* value
GSTT1 (+) GSTM1 (−)	30 (24%)	54 (43.2%)	Ref. 1.00		Ref. 1.00		
GSTT1 (−) GSTM1 (+)	20 (16%)	15 (12%)	0.41 (0.1864 to 0.9316)		0.62 (0.4165 to 0.9378)		0.003

Abbreviations: GST: glutathione S-transferase.

**Table 4:** Association of VEGF insertion/deletion (I/D) polymorphism of 18 bp fragment at –2549 position of the promoter region in VEGF gene in PCOS cases and controls.

Subjects	N=	I	ID	D	Df	X^2^	I	D	*p*-Value
Cases	125	32(25.5%)	50(40%)	43(32%)	2	11.38	0.58	0.52	0.0034
Controls	125	38(30.4%)	67(53.6%)	20(16%)			0.65	0.35	
Association of VEGF-rs699947 C>A gene variation in PCOS cases and controls									
Subjects	N=	CC	CA	AA	Df	X^2^	C	A	*p* value
Cases	125	45(36%)	54(43.2%)	26(20.8%)	2	7.58	0.58	0.42	0.020
Controls	125	63(50.4%)	49(39.2%)	13(10.4%)			0.70	0.30	

**Table 5:** Association of VEGF insertion/deletion (I/D) polymorphism of 18 bp fragment at –2549 position of the promoter region in VEGF gene in SCD cases and controls.

Genotypes	Controls	PCOS Cases	OR (95% CI)	Risk Ratio (RR)	*p*-Value
	(N = 125)	(N = 125)			
Codominant inheritance model					
VEGF-(II)	38	32	1 (ref.)	1 (ref.)	
VEGF-(ID)	67	50	0.88 (0.4883 to 1.6084)	0.94 (0.7266 to 1.2368)	0.69
VEGF-(DD)	20	43	2.55 (1.2566 to 5.1874)	1.71 (1.1223 to 2.6054)	0.009
Dominant inheritance model					
VEGF-(II)	38	32	1 (ref.)	1 (ref.)	
VEGF-(ID+DD)	87	93	1.26 (0.7297 to 2.2084)	1.12 (0.8636 to 1.4606)	0.390
Recessive inheritance model					
VEGF-(I+ID)	105	82	1 (ref.)	1 (ref.)	
VEGF-(D)	20	43	2.75 (1.5048 to 5.0367)	1.76 (1.2052 to 2.5957)	0.001
Additive inheritance model (Allele)					
VEGF-(I)	143	112	1 (ref.)	1 (ref.)	
VEGF-(D)	107	136	1.62 (1.1390 to 2.3121)	1.27 (1.0653 to 1.5226)	0.007

**Table 6:** Association of VEGF rs699947 C>A genotypes to predict the risk of PCOS susceptibility.

Genotypes	Healthy Controls	PCOS Cases	OR (95% CI)	Risk Ratio (RR)	*p*-Value
	(n = 125)	(n = 125)			
Codominant inheritance model					
VEGF-(CC)	63	45	1 (ref.)	1 (ref.)	
VEGF-(CA)	49	54	1.54 (0.8955 to 2.6580)	1.22 (0.947 to 1.586)	0.11
VEGF-(AA)	13	26	2.80 (1.2992 to 6.0344)	1.75 (1.092 to 2.804)	0.008 *
Dominant inheritance model					
VEGF-(CC)	63	45	1 (ref.)	1 (ref.)	
VEGF-(CA + AA)	62	80	1.80 (1.0888 to 2.9971)	1.33 (1.0451 to 1.7079)	0.022 *
Recessive inheritance model					
VEGF-(CC + CA)	112	99	1 (ref.)	1 (ref.)	
VEGF-(AA)	13	26	2.26 (1.1029 to 4.6418)	1.59 (1.0036 to 2.5266)	0.025 *
Additive inheritance model (Allele)					
VEGF-(C)	175	144	1 (ref.)	1 (ref.)	
VEGF-(A)	75	106	1.71 (1.1877 to 2.483)	1.32 (1.0842 to 1.6167)	0.004 *

*—indicate significant *p* value.

## Data Availability

All data connected with the research study are presented in the manuscript.
